# Identification of candidate genes for devil facial tumour disease tumourigenesis

**DOI:** 10.1038/s41598-017-08908-9

**Published:** 2017-08-18

**Authors:** Robyn L. Taylor, Yiru Zhang, Jennifer P. Schöning, Janine E. Deakin

**Affiliations:** 1 School of Biological Sciences, University of Tasmania, Hobart, Tasmania 7001 Australia; 2 Research School of Biology, The Australian National University, Canberra, ACT 2601 Australia; 3 Institute for Applied Ecology, University of Canberra, Canberra, ACT 2617 Australia; 4 Australian Institute for Bioengineering and Nanotechnology, University of Queensland, Queensland, 4067 Australia

## Abstract

Devil facial tumour (DFT) disease, a transmissible cancer where the infectious agent is the tumour itself, has caused a dramatic decrease in Tasmanian devil numbers in the wild. The purpose of this study was to take a candidate gene/pathway approach to identify potentially perturbed genes or pathways in DFT. A fusion of chromosome 1 and X is posited as the initial event leading to the development of DFT, with the rearranged chromosome 1 material now stably maintained as the tumour spreads through the population. This hypothesis makes chromosome 1 a prime chromosome on which to search for mutations involved in tumourigenesis. As DFT1 has a Schwann cell origin, we selected genes commonly implicated in tumour pathways in human nerve cancers, or cancers more generally, to determine whether they were rearranged in DFT1, and mapped them using molecular cytogenetics. Many cancer-related genes were rearranged, such as the region containing the tumour suppressor *NF2* and a copy gain for *ERBB3*, a member of the epidermal growth factor receptor family of receptor tyrosine kinases implicated in proliferation and invasion of tumours in humans. Our mapping results have provided strong candidates not previously detected by sequencing DFT1 genomes.

## Introduction

Tasmanian devils (*Sarcophilus harrisii*) are the world’s largest marsupial carnivores endemic to the island state of Tasmania, Australia. Wild Tasmanian devil populations have been succumbing to a clonal metastatic transmissible cancer known as Devil Facial Tumour Disease (DFTD), which was first noticed in the mid 1990s in the North East corner of the state and has since spread rapidly across the wild devil population range of the island, dramatically reducing Tasmanian devil numbers and causing devils to be listed as an endangered species in 2009^1, 2^.

DFTD is a soft tissue neoplasm of Schwann cell origin^3, 4^, often appearing within oral cavities and around the jowls^2, 5^ that, so far, has no known pathogenic etiology or transmission route. DFTD is most likely transmitted allogeneically where free live tumour cells metastasise from affected individuals to healthy individuals through intimate contact such as biting, which is a social behaviour in groups of devils^6–9^. It has been shown that the DFT cells are not recognised by the devil immune system as ‘non-self’ and evasion of the immune system allows free live tumour cells to grow rapidly and metastasise^10, 11^.

**Figure 1:**
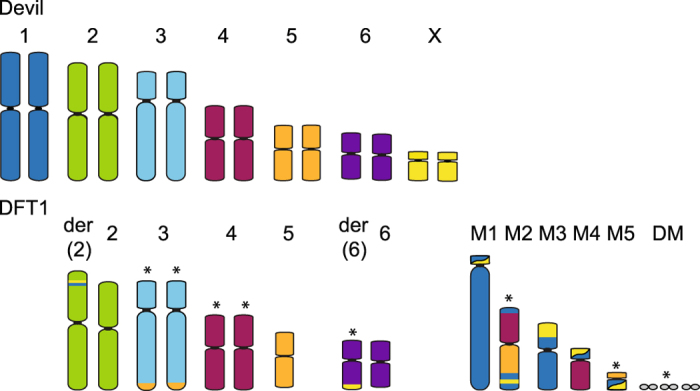
The normal devil karyotype consists of 6 pairs of autosomes and a pair of sex chromosomes. In DFT1, chromosome 1 and X material is spread across up to 5 marker chromosomes (M1-M5) (colour-coded to show their homology to devil chromosomes). Chromosomes varying between and within DFT1 strains are indicated by an asterisk, including the presence or absence of double minutes (DM). The karyotype has been arranged according to the original devil and standard dasyurid karyotypes^46^, differing from the karyotype presented by Pearse and Swift^6^ in the order of chromosomes 1 and 2.

Allogeneic transmission of clonal tumour cells, cells originating in one individual and continue passaging throughout different individuals and populations, accumulating genetic mutations over time, was suggested as the method of transmission following the observation that the karyotype of devil facial tumour (DFT) cells from different individuals were the same^6, 12^. The chromosomes from DFT cells were highly rearranged, with both copies of chromosome 1 and X and one copy of chromosome 5 rearranged to form distinctive marker chromosomes^13^ (Fig. 1). Genotyping of microsatellites, single nucleotide polymorphisms (SNPs) and major histocompatibility complex (MHC) alleles as well as mitochondrial DNA sequencing have supported this theory of allogenic transmission, demonstrating that tumour genotypes did not match those of the their host and genotypes of tumours from different individuals were identical or near identical to each other^3, 14–16^. A second, independently derived, transmissible tumour (DFT2) causing DFTD has been recently described in animals from the south east of Tasmania but it has a very different karyotype to the first tumour (DFT1) and most likely a different cellular origin^17^.

DFT1 is proposed to have arisen from telomeres on one homologue of chromosomes 1 and X becoming critically short, permitting the fusion of these two chromosomes, followed by a series of breakage-fusion-bridge cycles to ultimately result in their shattered appearance in the DFT1 karyotype^13, 18^. The transmissible tumour is evolving as it as spreads through the devil population, accumulating mutations resulting in visible, karyotypic differences^12^. Four different karyotypes have been classified based on G-banding and are referred to as Strains 1 to 4 of DFT1. Strains 1 and 2 are the most common strains detected across Tasmania and differ by the presence of an additional marker chromosome in Strain 2; Strain 3 is geographically restricted to the Forrestier Peninsula; Strain 4, the most derived and potentially more virulent strain, is restricted to eastern Tasmania^12^. The use of these classifications into strains may be misleading as similar magnitude differences have been detected within strains as between strains when more sensitive, molecular cytogenetic analyses were performed^13^. The karyotypic differences appear mainly in material from chromosomes 3, 4 and 5^12, 13^ (Fig. 1) but the arrangement of chromosome 1 material in the tumour is stable, suggesting that this arrangement is required for the survival of the tumour^13^.

Mutations driving tumourigenesis in DFTD are yet to be identified. The emergence of a second transmissible tumour makes it imperative that we gain a better understanding of DFT tumourigenesis. It is challenging to determine mutations contributing to tumorigenesis in human cancers, where there is access to matched tumour and host samples and extensive resources, including a well-assembled and annotated human reference genome^19^. In DFTD, extra layers of difficulty exist for determining genes involved in tumourigenesis. Transcriptome sequencing has determined a Schwann cell origin of DFT1^3^, but identifying genes aberrantly expressed in the tumour is limited by the lack of a Schwann cell control to compare with DFT1 expression. Attempts to isolate and establish devil Schwann cell cultures have proven challenging (R. Taylor, unpublished). Moreover, two independent genome projects have sequenced devil and DFT1 genomes, and have only detected a very small number of sequence mutations in genes that could potentially contribute to DFT1 tumourigenesis but no obvious driver candidates^15, 16^. Furthermore, the lack of a sample from the founder animal in which the tumour initially arose leaves some doubt as to whether sequence differences are mutations that may affect gene function or merely polymorphisms between different individuals. Considering the complex chromosome rearrangements in DFT1^13^ and the known role chromosome rearrangements can play in tumorigenesis in human cancers^20^, structural mutations may play a prominent role in DFT1 tumourigenesis. Unfortunately, the current reference devil genome assembly is fragmented (over 35,000 scaffolds), making it challenging to reliably detect rearrangements, although some rearrangements have been detected and validated^15^.

As an alternative approach, we have exploited our understanding of the structural changes and the Schwann cell origin of the tumour to predict prime candidate genes that may be affected in DFT1, in order to identify potentially perturbed pathways for further investigation. The fusion of chromosome 1 and X is predicted to be the event leading to tumourigenesis in DFT1^13, 18^, making the large chromosome 1 a logical place to start a search for potential driver mutations, with strong candidate genes being those involved in tumourigenesis in peripheral nerve sheath tumours in humans. Firstly, we decided to test the stability of this chromosome through further mapping of bacterial artificial chromosome (BAC) clones on DFT1 chromosomes from three different samples for which there is already mapping data available^13^. Secondly, we selected cancer-related genes located on the other autosomes. We predicted that genes located on devil chromosome 1 and 5 would be rearranged in DFT1 as these chromosomes are highly rearranged in the tumour, and genes on devil chromosomes 2 and 6 would not be perturbed as these chromosomes have few rearrangements^13^. Some regions of chromosome 4 are rearranged in DFT1^13^, so we predicted that it was possible that at least some of the cancer-related genes we selected from this chromosome would be rearranged in the tumour. Our approach has given further support for the stable nature of chromosome 1 material in DFT1 and provided a means for selecting genes and pathways for further analysis for their role in DFT1 tumourigenesis.

## Results

We tested our hypothesis that the rearranged chromosome 1 material in DFT1 is stable and therefore, a likely chromosome to harbour mutations driving DFT1 tumourigenesis. We generated a dense map of devil chromosome 1 by mapping an additional 29 BACs to devil chromosome 1 and three DFT1 samples, with distinct karyotypic differences^13^ and from different geographic locations (Supplementary Figure S1). Then, we mapped 22 cancer-related genes to chromosome 1 and other autosomes to identify potentially perturbed genes/pathways in DFT1.

### Dense cytogenetic map of chromosome 1 BACs on normal and DFT1 chromosomes

**Figure 2:**
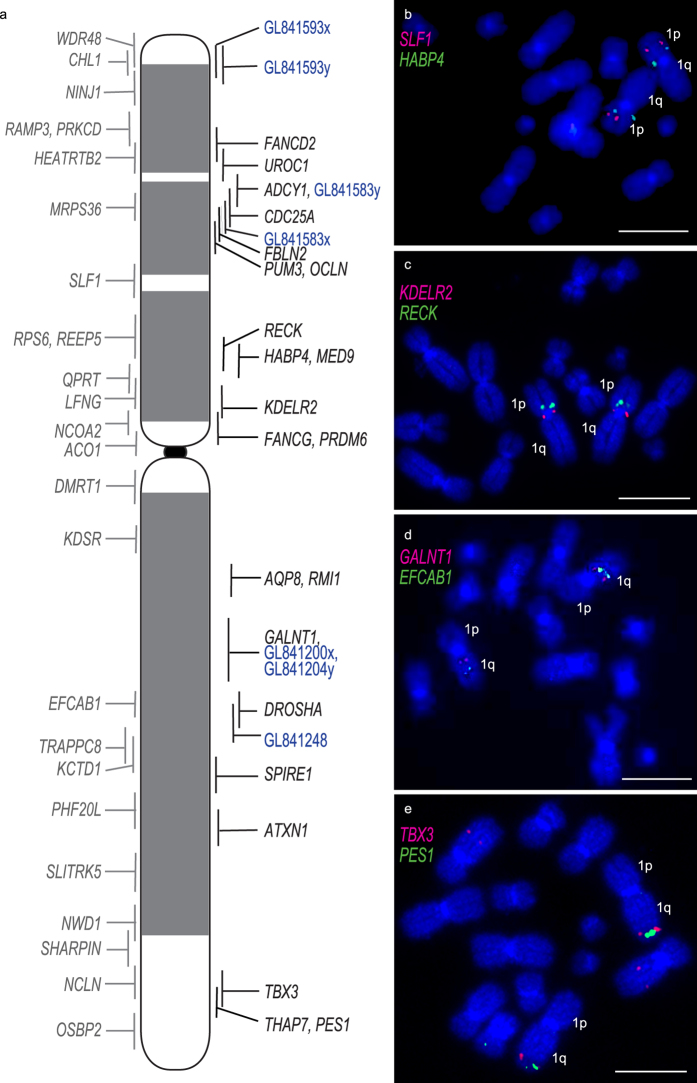
(**a**) Map of normal devil chromosome 1, including genes from the first generation map^13^ shown to the left of the chromosome, genes mapped as part of this study and large sequence scaffolds (blue) are shown on the right of the chromosome. Vertical lines indicate the Flpter value (±1 standard deviation). Shading on the devil chromosome corresponds to DAPI banding. (**b**–**e**) Examples of FISH results for genes mapping close together on chromosome 1. Scale bars represent 10 μm.

The devil genome project, which sequenced flow sorted chromosomes, allowed genes to be assigned to normal devil chromosomes but their precise location on the chromosome was unknown^15^. We isolated BAC clones for mapping corresponding to genes previously mapped to tammar wallaby chromosomes^21^ (to permit a direct comparison of gene arrangement between marsupials if needed), or the ends of large (>3 Mbp) sequence scaffolds in the devil genome assembly (version 7.0). The genome coordinates of most BAC clones within the devil genome assembly were determined by sequencing the ends of each BAC clone (Supplementary Dataset 1A). The position of the 29 devil BAC clones on devil metaphase chromosomes was incorporated into the previous version of the cytogenetic map for chromosome 1^13^ by a combination of ordering genes on the chromosome based on Flpter (fractional length p arm terminus) values^22^ and further refined with two-colour FISH experiments for genes mapping to similar locations, resulting in a map consisting of 53 BAC clones distributed across the chromosome (Fig. 2).

**Figure 3:**
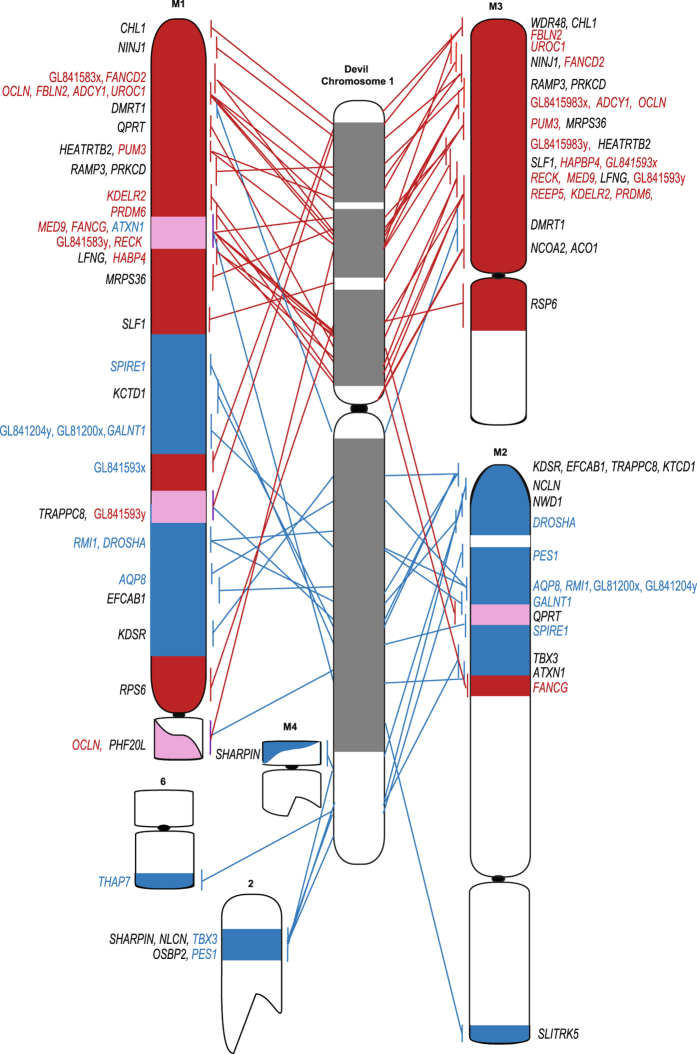
Comparison of consensus mapping data from three DFT1 strains with the location of genes on devil chromosome 1. Lines indicate the location of the genes on the normal devil chromosome 1 (DAPI banded chromosome indicated in the centre) compared to their location on DFT1 chromosomes. Regions of DFT1 chromosomes and lines indicating position of genes have been colour-coded red for devil 1p genes and blue for devil 1q genes. Regions containing genes from both chromosome 1 arms are indicated in pink. Genes mapped as part of this study are indicated in colour. Genes mapped previously^13^ are in black.

We then used DFT1 samples that had been used previously to construct the original cytogenetic map of the DFT1 tumours^13^ and compared gene locations between devil chromosome 1 and all three geographically isolated tumours (Supplementary Figure S1). We detected no differences in the location of BACs between three different samples (Supplementary Dataset 1B). A comparison of the gene arrangement on devil chromosome 1 versus the combined data for the DFT1 arrangement demonstrates the highly rearranged nature of chromosome 1 DNA on marker chromosome 1 (M1) (Fig. 3). The other copy of chromosome 1 appears to have essentially split at the centromere (with only a few exceptions), with 1p material located on M3 and 1q DNA on M2.

### Mapping cancer-related genes in DFT1

We selected genes commonly implicated in tumour pathways in human malignant peripheral nerve sheath tumours (MPNSTs) or schwannomas (tumours of Schwann cells), as well as some genes often generally perturbed in human cancers, to determine if they were rearranged in DFT1 and therefore, candidates for contributing to DFT1 tumourigenesis (Table 1; Supplementary Dataset 1C). Based on previous molecular cytogenetic characterisation of devil and DFT1 genomes^13^, and genome sequence information^15^, we were able to predict which genes were most likely to be rearranged in devil facial tumours and those unlikely to be affected by structural mutations. Our mapping data confirmed the chromosome assignment given in the genome assembly, localised candidate genes to specific sites on devil and DFT1 chromosomes and allowed us to test our predictions concerning the genes likely to be rearranged in DFT1. BACs were mapped to at least two DFT1 samples.

**Table 1:** Selected cancer-related genes.

Gene	Role in Schwannomas/MPNSTs	Devil Chromosomal location	DFT1 Chromosomal location
*ATRIP*	Component of DNA damage checkpoint	1p	M1q and M3q
*EGFR*	Associated with development of tumours derived from Schwann cells^47, 48^.	1p	M1q and M3q
*IGFBP1*	Regulates proliferation, adhesion and survival in schwannomas^49^.	1p	M1q and M3q
*CDKN2A*	Mutated in 50% of MPNSTs, dysregulating p16INK4A cyclinD1-cyclin-dependent kinase (CDK)4-Retinoblastoma (Rb) and/or p19ARF -Mdm2-p53 cell cycle regulatory pathways^50–52^.	1q	M1q and M3q
*ICAM1*	Involved in tumour growth and metastatis	1q	der(2p) and M2q
*TERT*	Telomerase lengthens telomeres in DNA strands (chromosomes) allowing senescent cells to become potentially immortal.	1q	M1q and M3p
*NF2*	Loss of this tumour suppressor is key to the development of most schwannomas^28^.	1q*	Region rearranged *THAP7* end: der(6q) *PES1* end: der (2p) and M2q
*LZTR1*	Loss of this tumour suppressor associated with schwannomas^35^.	1q	Rearranged, near breakpoint
*DICER1*	Involved in microRNA biogenesis with perturbations linked to human cancers^53^	2p	2p, der(2p) Amplification on double minutes in sample 11.3178
*IGF1R*	Frequently amplified in MPNSTs^54^.	2p	2p, der(2p) (unchanged)
*ATMIN*	Regulates checkpoint kinase ATM^55^	2q	2p, der(2p) (unchanged)
*KCNH6*	Expression frequently perturbed in human cancers^56^	4p	4p, 4p and M4q (copy gain)
*NGF*	Promotes neuronal cell survival and differentiation particularly in Schwann cells^57^.	4p	4p, 4p and M4q (copy gain)
*NRAS*	Proto-oncogene	4p	4p, 4p and M4q (copy gain)
*NF1*	A negative regulator of the ras signalling pathway, often mutated in MPNSTs^28^.	4p*	4p, 4p and M4q (copy gain)
*IGF2R*	Involved in multiple cellular processes dysregulated in tumours^58^	4q	4q, 4q (unchanged)
*TP53*	Regulates the cell cycle and is known as the “guardian of the genome” preventing genomic mutations. Mutated in 75% of MPNSTs^36, 52^.	4q	4q, 4q (unchanged in most samples) 4q, 4q, M1p, M5p (copy gain in sample 06.2109)
*ERBB3*	A member of the epidermal growth factor receptor family and is over expressed in many cancers including schwannommas and MPNSTs.	5p	4p, 4p, M4q (copy gain and rearrangement)
*GALNT2*	Associated with tumour invasion	5p	4p, 5p, M2p (copy gain and rearrangement)
*IGF1*	Growth factor	5p	5p, M2p
*MDM2*	Amplified in a portion of MPNSTs^59^.	5p	5p, M2p
*EGF*	Promotes cell migration in MPNSTs^39^.	6p	2p, der(2p) (translocation)

^*^ *NF1* and *NF2* were not directly mapped.

**Figure 4:**
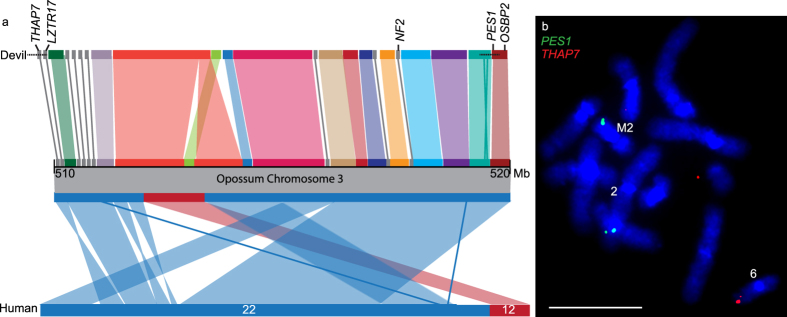
The *NF2* region (**a**) Comparison of the 10 Mb region surrounding *NF2* on opossum chromosome 3 (position 510–520 Mb) with devil reference genome scaffolds (each scaffold is a different colour) and human chromosomes 22 (blue) and 12 (red). The order of the devil scaffolds is unknown, although BAC end sequences join several scaffolds indicated by a dotted line. (**b**) Rearrangement detected in the *NF2* region in DFT1. *PES1* is located on DFT1 derivative chromosome 2 and M2. Only one signal is detected for *THAP7/LZTR1*, localising to derivative chromosome 6. Scale bar represents 10 μm.

As predicted, genes located on chromosomes 1 and 5 were predominantly located on the rearranged derivative chromosomes (Table 1). One stand-out candidate gene identified to be on chromosome 1 from sequencing of flow sorted chromosomes^15^ was *NF2*, a tumour suppressor gene located on human chromosome 22q12.2. *NF2* has been implicated in a diverse range of nervous system tumours, including those with a Schwann cell origin^23^. Attempts to isolate BAC clones for this gene failed, despite multiple attempts with different overgo probes^24^ and two different devil BAC libraries, suggesting that this gene is not represented in either of the BAC libraries. However, we mapped two other BACs from the human 22q region, containing genes *THAP7* and *LZTR1* (VMRC49_433C15) and *PES1* (VMRC49_253L6). *OSBP2*, also from this region, was previously mapped to distal 1q^13^. We aligned the devil scaffolds to a 10 Mbp region of the opossum genome (the marsupial genome currently with the most contiguous assembly across this region) encompassing the *NF2* gene. BAC end sequence data joins some of the devil reference genome scaffolds in this region (Fig. 4a). The genes in this region map very close together on the distal end of devil 1q (Fig. 3). The *NF2* gene is the only gene annotated on the 199,584 bp scaffold, GL842060.1. Mapping of the genes corresponding to the 10 Mbp region surrounding opossum *NF2* (*THAP7*/*LZTR1*, to *PES1/OSBP2*) onto DFT1 chromosomes shows that this region has undergone rearrangement in DFT1. One copy of *PES1* and *OSPB2* are located on the derived chromosome 2. The other copy of *PES1* is located on marker chromosome 2 (M2). *THAP7/LZTR1* does not map to either of these chromosomes (Figs 3 and 4b). Instead, the *THAP7/LZTR1*-containing BAC is located only on the derivative 6q (i.e. one copy has been deleted). *OSBP2* has also previously been shown to have experienced a deletion of one copy and to only be present on the derivative chromosome 2 in DFT1^13^ (Fig. 3).

**Figure 5:**
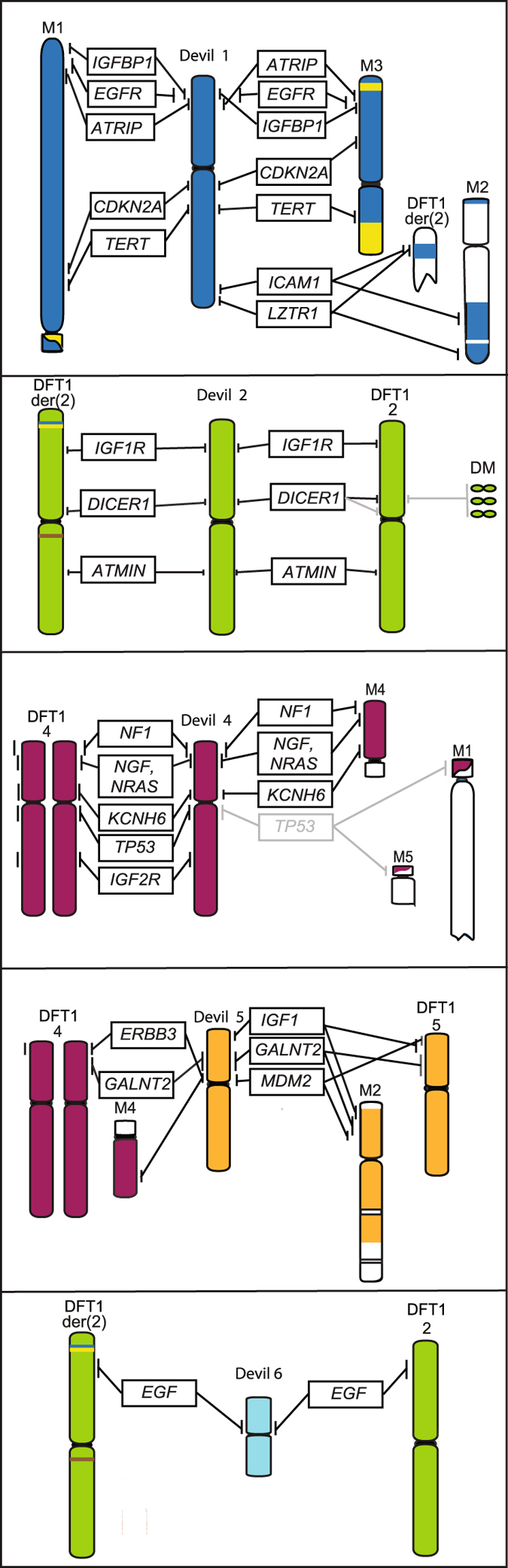
Location of cancer-related genes on devil and DFT1 chromosomes. Genes in grey indicate variations observed between samples.

All other genes selected from devil chromosome 1 were present in DFT1 in two copies, with some remaining in what appears to be a similar genomic context to what they are on devil chromosome 1, despite being located on marker chromosomes (Fig. 3). However, two of the four chromosome 5 genes have experienced a copy gain in DFT1; *ERBB3* is located on the short arm of both copies of chromosome 4, as well as the long arm of M4, and *GALNT2* is located on the short arm of one homologue of chromosome 4, the short arm of chromosome 5 and the short arm of M2 (Fig. 5).

**Figure 6:**
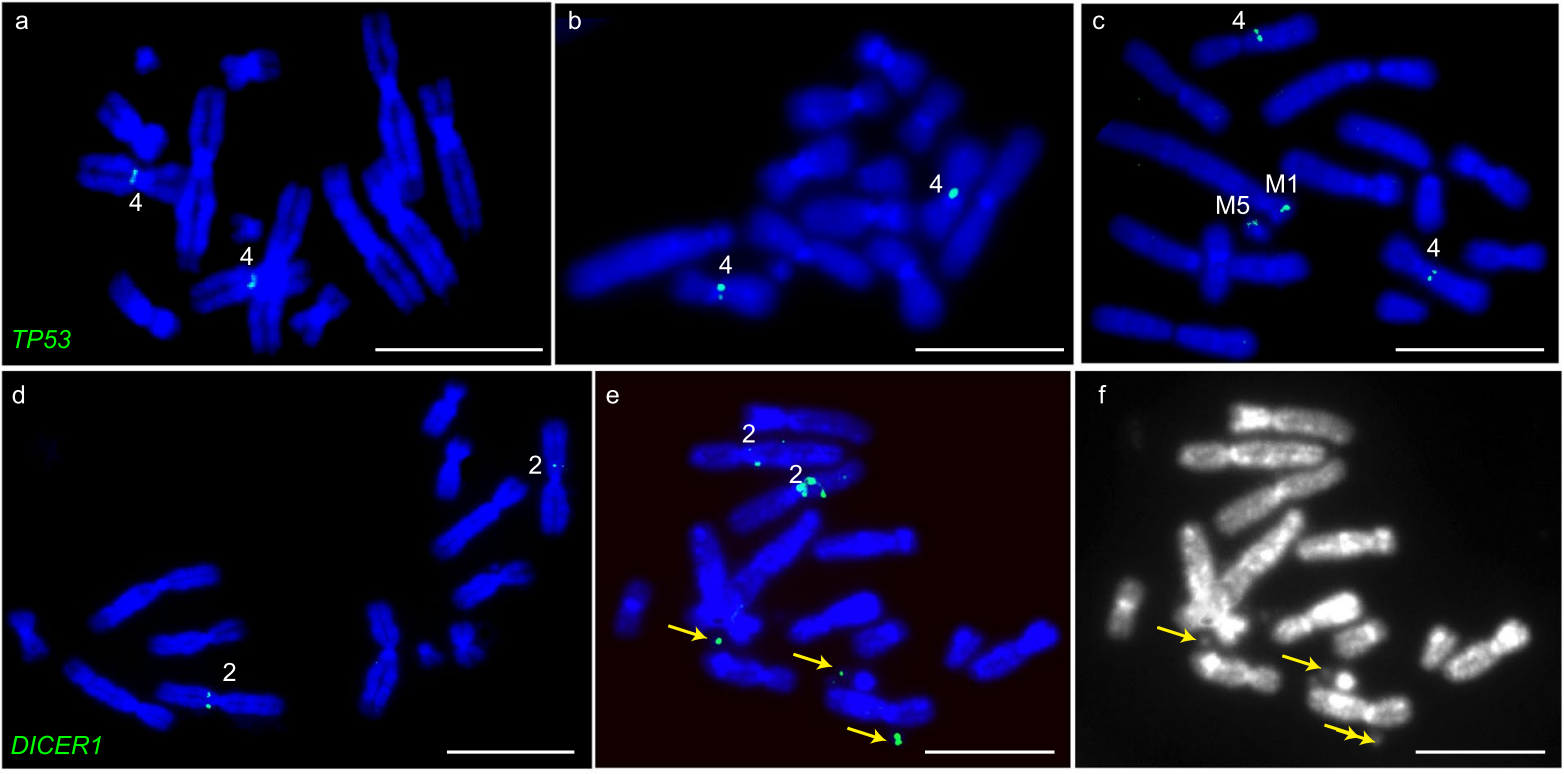
(**a**) *TP53* is located on chromosome 4 on normal devil chromosomes and (**b**) in DFT1 sample 06.1926. (**c**) *TP53* is located on two additional chromosomes (M1 and M5) in sample DFT1 06.2109. *DICER1* localises to (**d**) chromosome 2 on normal devil chromosomes. (**e**) In DFT1 sample 11.3178, *DICER1* is amplified on double minutes (indicated by arrows). (**f**) DAPI image to show that green fluorescent signals in (**e**) correspond to double minutes. Scale bars represent 10 μm.

We predicted genes on chromosome 4 would possibly be rearranged in DFT1. All six genes selected from this chromosome were located in the same positions on chromosome 4 in DFT1 as they are on normal devil chromosomes. However, three of these genes, including the “guardian of the genome” *TP53*, *NGF* and *KCNH6*, had at least one addition copy located on another chromosome (Fig. 5), although *TP53* only had additional copies in one sample (06.2109) tested (Fig. 6). Several attempts to isolate a BAC clone containing devil *NF1* using two different BAC library filters and different overgo probes failed, suggesting that it may not be represented in either of the two available devil BAC libraries (VMRC-49 and VMRC-50)^25^. However, we were able to isolate a BAC clone corresponding to the genome scaffold containing the *NF1* gene, which demonstrated that this region of the genome has also experienced a copy gain in DFT1 (Fig. 5).

Cancer-related genes from chromosomes 2 and 6 were also mapped to test our prediction that these were unlikely to be rearranged in DFT1. This prediction was correct for three chromosome 2 genes, which each mapped to the same location on chromosome 2 on normal devil and DFT1 chromosomes from most samples. The one exception was *DICER1*, which localised to two locations on chromosome 2 in sample 11.3178 and was amplified on double minutes (Fig. 6). Contrary to our prediction, *EGF*, the only gene mapped from devil chromosome 6, was not located on chromosome 6 but both copies mapped to chromosome 2 (Fig. 5).

## Discussion

It is challenging to determine mutations contributing to pathogenesis in human cancers, where there is access to extensive reference resources^19^. In DFT1, extra layers of difficulty exist for identifying mutations driving tumourigenesis. Considering the complex chromosome rearrangements in DFT1, structural mutations play a significant role in DFT1 pathogenesis but the current state of the devil reference genome assembly make the accurate identification of structural mutations challenging based solely on sequence data. By employing a molecular cytogenetic approach to target candidate genes, we have detected strong candidates for further investigation for a role in DFT1 pathogenesis. Of the 22 genes selected for mapping, those of particular interest for further investigation are those in a very different genomic context (e.g. located on a different chromosome) compared to the normal arrangement and/or show a change in copy number in the tumour common to all strains (Table 1).

The event leading to DFT1 tumourigenesis is proposed to be the fusion of chromosome 1 with an X chromosome, which led to a series of breakage-fusion-bridge cycles and extensive rearrangement of chromosome 1 material on M1^13, 18^. With this idea in mind, along with the now seemingly stable nature of the extensively rearranged chromosome 1 material in DFT1, chromosome 1 is a likely source for DFT1 driver mutations. Although many genes have been implicated with cancer, we chose candidates based on their known association with nerve cell neoplasms or being commonly involved in cancers more generally. The strongest candidate on devil chromosome 1 is *NF2*. This hypothesis is based on the frequency of breakpoints in the region encompassing *NF2* in humans (corresponding to 22q11-13) involved in MPNSTs^26, 27^, and the role of the loss or inactivation of *NF2* in Schwann cell tumours^28^. Although we were unable to isolate and map the *NF2* gene as part of this study, we demonstrated that this region of the DFT1 genome has undergone rearrangement and even some deletion, including the deletion of one copy of the tumour suppressor *LZTR1* ^29^ (Fig. 4b).

**Figure 7:**
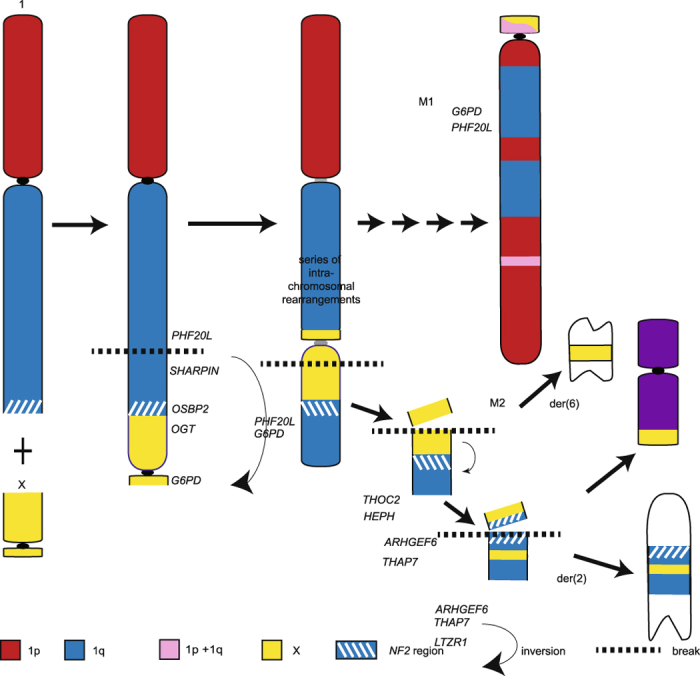
Proposed scenario for the fusion of chromosome 1 and X leading to the formation of marker chromosome 1. A loss of telomeres from 1q (adjacent to the *NF2* region) permitted the fusion with the X chromosome, which had also experienced telomere loss. The resulting dicentric chromosome broke between the *PHF20L* and *SHARPIN* genes, permitting an inversion of region around the 1q/X fusion. A further break led to the region around the original 1q/X fusion containing the *NF2* region b﻿reaking away from M1. After a break permitting the translocation of the fragment containing *THOC2* and *HEPH* to M2, a further inversion around the *NF2* region brought *THAP7* from 1q and *ARHGEF6* from Xq together. These genes then translocated to derivative chromosome 6 whereas the remaining segment translocated to derivative chromosome 2.

The additional mapping of chromosome 1 genes on devil and DFT1 chromosomes enabled a scenario for the initial chromosome rearrangements contributing to DFT1 tumourigenesis to be proposed (Fig. 7). Previous research has suggested the loss of telomeres led to the fusion of chromosomes 1 and X^13, 18^. Our mapping data suggest the initial fusion most likely occurred close to the *NF2* region at the distal end of 1q and the distal end of Xq. Therefore, the *NF2* region would have been close to the original site of genome instability and a site of further instability, with this region being separated from the rest of chromosome (translocated to chromosome 2 and 6). The other copy of this region, located on chromosome M2 has also experienced rearrangement and even deletion (*OSBP2*, *THAP7/LZTR1*).

*NF2* is a tumour suppressor involved in regulating intracellular signalling pathways and cell cycle progression^30^. In schwannoma cells lacking *NF2*, epidermal growth factor receptor tyrosine kinases ErbB2, ErbB3 and EFGR are overabundant on the cell surface, upregulating their downstream targets, thereby driving cell proliferation^31–33^. This is particularly interesting in light of the mapping of *ERBB3* in DFT1. *ERBB3* is in a very different genomic context in the tumour (located on chromosome 4 rather than 5) and has experienced a copy number gain (Fig. 5). Consistent with the Schwann cell origin of DFT1^3^, it is therefore not surprising to find *ERBB3* and potentially *NF2* the subject of structural mutations. NF2, ErbB3 and downstream effectors PI3K-Akt or MAPK signalling pathways are therefore prime candidates for future immunohistochemistry and functional studies to determine if they are involved in DFT1 tumourigenesis. It has recently been shown that elevated levels of ErbB3 are detectable in devil sera several months prior to visual tumour eruptions^34^ making it imperative that the pathway involving NF2 and ErbB3 be prioritised for further investigation. Additionally, germline mutations in the tumour suppressor *LZTR1* are associated with a predisposition for an inherited disorder responsible for the development of multiple schwannomas in humans^35^, making the entire *NF2* region a high priority for further investigation.

*TP53* is commonly referred to as “the guardian of the genome” because of its central role as a cell cycle checkpoint and tumour suppressor. It is commonly mutated in many cancers, including approximately 75% of human MPNSTs, where mutations commonly result in deletion or loss of function of *TP53* ^36^. It has only experienced a copy gain in one of the samples tested (06.2109) (Fig. 6), which is interesting considering most MPNST mutations involve a p53 loss and raises questions as to whether this copy gain affects *TP53* function in this strain. Transgenic mice with three copies of *Trp53* had an enhanced response to DNA damage and an increased resistance to cancer^37^. It seems likely that *TP53* function has been affected in DFT1 since there are four copies of this gene. If appropriately regulated, these additional copies should enhance the role of TP53 in tumour suppression. However, two of these *TP53* copies are in a completely different genomic context, being located on chromosomes M1 and M3, and may be aberrantly regulated.

Genome sequencing has previously failed to detect changes in copy number for chromosome 4, making the gain in copy number of *TP53*, the *NF1* scaffold, *KCNH6* and *NGF* (Fig. 5) an unexpected finding, whereas copy number gains for chromosome 5 had been previously reported^15^. Among the chromosome 4 genes, Neurofibromin 1 (*NF1*) was the top candidate prior to mapping. *NF1* is a tumour suppressor gene, where loss of function mutations are associated in humans with the autosomal dominant syndrome Neurofibromatosis type 1. Patients with this syndrome often develop MPNSTs, many of which are of Schwann cell origin^38^. The loss of *NF1* function is seen as an early step in the development of neurofibromas, which then may progress to MPNST with mutations in other tumour suppressor genes such as *TP53* and *CDKN2A* ^28^. In this context, a copy gain for the *NF1* region rather than a deletion, make it less likely that *NF1* is a driver of DFT1 tumourigenesis. In additional, the extra copy of this region is also located on chromosome M4, a highly methylated chromosome compared to other DFT1 chromosomes^18^, which may be an indicator of transcriptional silencing of this chromosome and therefore, may not alter the overall gene expression of genes on the *NF1* scaffold. The copy number gains observed for *NGF* and *KCNH6* also have a low probability of being driver mutations of DFT1 as they are not on a chromosome posited to be initially involved in the formation of the tumour. They also have two copies of the gene in their normal location. Like the *NF1* scaffold, the additional copy of *KNCH6* is located on chromosome M4. The additional copy of *NGF* is located chromosome M2 and may be aberrantly expressed.

The translocation of *EGF* (epidermal growth factor) from chromosome 6 to chromosome 2 in DFT1 was surprising, as rearrangements involving the movement of genes from chromosome 6 had not been previously detected. Finding both copies in the same location on chromosome 2 is intriguing and suggests that this region of chromosome 2 may have undergone repair to incorporate *EGF* on both chromosome 2 homologues. In MPNSTs, EGF promotes tumour migration^39^, making it another candidate for functional analysis.

The amplification of *DICER1* on DFT1 double minutes in tumour 11.3178 (Fig. 4) may explain the anecdotal evidence that this is a more aggressive lineage (strain 4) of DFT1, killing animals more quickly than other strains and resulting in a reduced transmission rate^12^. *DICER1* is a key gene involved in the biogenesis of microRNAs. The amplification of this gene may provide an explanation for the faster growth rate observed for strain 4 and its presumed more aggressive nature^12^. Changes in the expression levels of *DICER1*, either an increase or decrease, are associated with aggressive human cancers^40^. In particular, overexpression of *DICER1* observed in human tumours has been associated with enhanced cell proliferation^41^ and poor prognosis^42^. It would be interesting to determine whether the amplification of *DICER1* correlates with an increase in *DICER1* expression and/or determine the effect of amplification on *DICER1* function.

## Conclusions

We have confirmed the stable nature of chromosome 1 material in DFT1, supporting the hypothesis that the highly rearranged arrangement of chromosome 1 material is required to drive the tumour^13^. We have also determined the location of cancer-related genes on DFT1 chromosomes and identified a key genomic region and pathways that are potentially perturbed in the tumour, warranting future analysis. This candidate gene approach has provided an alternative strategy for identifying genes contributing to DFT1 tumourigenesis in a non-traditional model species, with a fragmented reference genome assembly^15^ and lacking other critical resources. Future research into rearrangements in DFT1 would benefit from an improved reference devil genome assembly and deep tumour sequencing. Such an improvement to the reference assembly is now within reach given the recent advances in sequencing approaches, such as high-throughput conformation capture sequencing, capable of producing more contiguous genome assemblies from physical contact information^43^. Comparison of a chromosome-level devil reference genome to devil facial tumour genomes would provide details of structural mutations at the base pair level and could be particularly powerful if combined with validation of rearrangements using molecular cytogenetics. Furthermore, it is imperative that a concerted effort be made to establish devil Schwann cell lines, which will be essential for the next steps to determine whether the functions of the prime candidate genes and/or regions (e.g. *NF2* region, including *LZTR1*, and *ERBB3*) are contributing to DFT1 tumourigenesis. Future studies are required to determine whether the transcript and/or proteins of genes occurring in rearranged regions are perturbed, followed by *in vitro* experiments on devil Schwann cells and DFT1 cells. There will undoubtedly be a suite of genes involved in DFT1 tumourigenesis but those identified here provide a good starting place for a more in-depth understanding of the drivers of DFT1.

## Methods

### BAC library screening

Overgo probes for (i) genes mapped in the tammar wallaby and located near the ends of human-opossum evolutionary conserved blocks^21^, (ii) sequences located near the ends of large sequence scaffolds in the devil genome assembly^15^ or (iii) for genes involved in human cancer. Devil genomic sequence for the genes or regions of interest were obtained from Ensembl (genome assembly Devil_ref7.0) and used as input sequence for the Overgo Maker program developed by The Genome Institute at Washington University. BLASTN searches of the devil genome assembly were performed with the resulting 40 bp overgo probe in order to confirm the specificity of all probes. Only probes with a single match across the entire length of the probe were used for BAC library screening to avoid hybridization to paralogous sequences. All overgos used for screening are listed in Dataset 1. BAC library filters were screened with pools of overgos, radioactively labelled as described by Ross *et al*.^24^, with dot blots performed on the resulting positive BACs in order to determine the gene each BAC contained following the protocol described by Deakin *et al*.^44^. In cases where overgos for genes failed to isolate a BAC for the target gene, we screened the library with different overgos for a location elsewhere on the sequenced genome scaffold.

BACs were end sequenced by Macrogen (Korea) using primers pCC1™/pEpiFOS™ forward sequencing primer (5′ GGATGTGCTGCAAGGCGATTAAGTTGG 3′) and pCC1™/pEpiFOS™ reverse sequencing Primer (5′ CTCGTATGTTGTGTGGAATTGTGAGC 3′). BAC end sequences were submitted to the GSS database (dbGSS Accession KS520996 - KS521086). BAC clones were confirmed to contain the gene of interest by BLASTN searching the devil genome assembly (Devil_ref v7.0), using the BAC end sequences as queries.

### Fluorescence *in situ* hybridization (FISH)

Devil metaphase chromosomes were prepared from a fibroblast cell line using standard methods previously described^12^. Blood and DFT1 samples were collected under approval of the Tasmanian Department of Primary Industries and Water Animal Ethics Committee and all methods were performed according to the relevant guidelines and regulations. Metaphase chromosomes were prepared by the Department of Primary Industries, Parks, Water and the Environment as previously described^12^.

DNA was extracted from 15 ml BAC clone cultures using the WIZARD Plus SV Minipreps DNA Purification System (Promega, Alexandria, NSW, Australia) using a slightly modified protocol; the volume of all reagents added prior to centrifugation to remove cell debris from the lysis process were doubled. Between 500 ng and 1 μg of DNA for each BAC was labelled by nick translation with either SpectrumGreen dUTP or SpectrumOrange dUTP (Abbott Molecular Inc., Des Plaines, IL, USA) and hybridized to normal devil or DFT chromosomes following the method described by Alsop *et al*.^45^. Unbound probe was washed off slides, counterstained and mounted as described by Deakin *et al*.^13^. Chromosomes and BAC signals were visualized and captured using either a Zeiss Axioplan2 epifluorescence microscope (Carl Zeiss Ltd, Cambridge, UK) fitted with a SPOT RT3 Monochrome charge coupled device (CCD) camera (Diagnostic Instruments Inc.) or Zeiss Axiocam MRm Scope A1 epifluorescence microscope fitted with an AxioCam Mrm Rev.3 CCD camera (Carl Zeiss Ltd). Measurements were taken to determine Flpter values using Adobe Photoshop software.

### Data availability

The datasets generated and/or analysed during the current study are available in the dbGSS repository (accession KS520996 - KS521086) or are included in this manuscript (and its Supplementary Information files).

## Electronic supplementary material

Supplementary Figure S1

Dataset 1
