# Supplementary material for: Identification of candidate genes for devil facial tumour disease tumourigenesis
Source: Sci Rep. 2017 Aug 18;7:8761. doi: 10.1038/s41598-017-08908-9 (PMC5562891; doi:10.1038/s41598-017-08908-9)
Supplement: Supplementary file 1 — Supplementary Figure S1 [file 41598_2017_8908_MOESM1_ESM.pdf]

Identification of candidate genes for devil facial tumour disease tumourigenesis

Robyn L. Talyor, Yiru Zhang, Jennifer P. Schöning, Janine E. Deakin

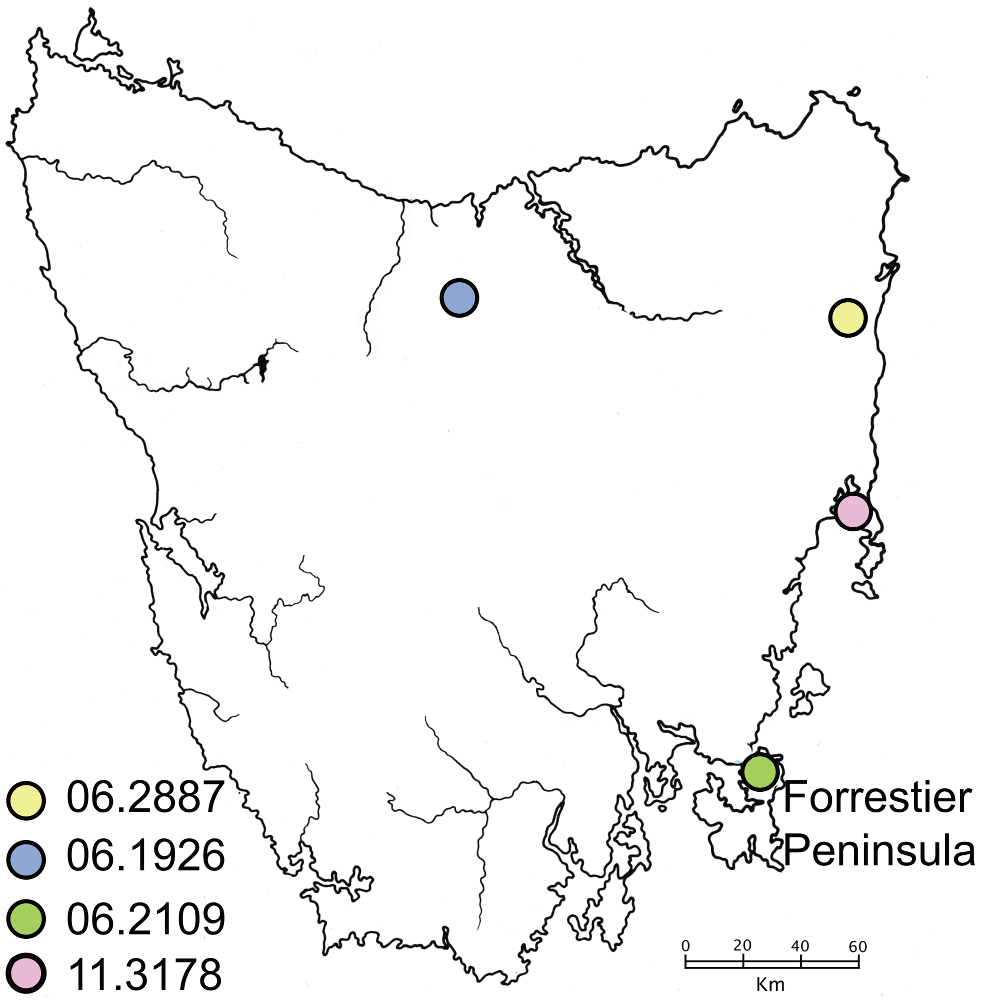

Supplementary Figure 1: The location within Tasmania of the four samples used in this study. This figure has been modified from Deakin JE, Bender HS, Pearse A-M, Rens W, O'Brien PCM, Ferguson-Smith MA, et al. (2012) Genomic Restructuring in the Tasmanian Devil Facial Tumour: Chromosome Painting and Gene Mapping Provide Clues to Evolution of a Transmissible Tumour. PLoS Genet 8(2): e1002483. https://doi.org/10.1371/journal.pgen.1002483 under the terms of the Creative Commons Attribution License (https://creativecommons.org/licenses/by/4.0/), which permits unrestricted use, distribution, and reproduction in any medium, provided the original author and source are credited.
